# The PHOENIX: Design and Development of a Three-Dimensional-Printed Drone Prototype and Corresponding Simulation Scenario Based on the Management of Cardiac Arrest

**DOI:** 10.7759/cureus.21594

**Published:** 2022-01-25

**Authors:** Bruno Gino, Kerry-Lynn Williams, Claire Siobhan Neilson, Philip d'Entremont, Adam Dubrowski, Tia S Renouf

**Affiliations:** 1 Emergency Medicine, Memorial University of Newfoundland, St. John's, CAN; 2 Faculty of Medicine, Memorial University of Newfoundland, St. John's, CAN; 3 Health Sciences, Ontario Tech University, Oshawa, CAN; 4 Emergency Medicine, Madrecor Hospital, Uberlândia, BRA; 5 Family Medicine, Memorial University of Newfoundland, St. John's, CAN; 6 General Medicine, University of Limerick, Limerick, IRL; 7 Research and Development, Ontario Tech University, Oshawa, CAN

**Keywords:** emergency medicine and trauma, three-dimensional (3d) printing, healthcare simulation, extracorporeal cardiopulmonary resuscitation, simulation based medial education, ‎3d printing, simulation in medical education, automatic external defibrillator, out of hospital cardiac arrest, drones

## Abstract

Sudden cardiac arrest (SCA) remains one of the most prevalent cardiovascular emergencies in the world. The development of international protocols and the use of accessible devices such as automated external defibrillators (AEDs) allowed for the standardization and organization of medical care related to SCA. When defibrillation is performed within five minutes of starting ventricular fibrillation (VF) and pulseless ventricular tachycardia (VT), the victim survival rate has increased considerably. Therefore, training healthcare professionals to use AEDs correctly is essential to improve patient outcomes and response time in the intervention. In this technical report, we advocate simulation-based education as a teaching methodology and an essential component of drone adaptation, novel technology, that can deliver AEDs to the site, as well as a training scenario to teach healthcare professionals how to operate the real-time communication components of drones and AEDs efficiently. Studies have suggested that simulation can be an effective way to train healthcare professionals. Through teaching methodology using simulation, training these audiences has the potential to reduce the response time to intervention, consequently, increasing the patient's chance of surviving.

## Introduction

Ischemic heart disease being the cause of sudden cardiac arrest (SCA) is one of the main causes of death in the world [1]. In 2013, in the United States, 63% of these events were out-of-hospital cardiac arrest (OHCA), with a survival rate of 9.5% [2]. This underscores the importance of high-quality cardiopulmonary resuscitation (CPR), public access to automated external defibrillators (AEDs), and postresuscitation care [2]. The use of drones, or unmanned aerial vehicles (UAVs), as instruments for medical use in a prehospital environment, has numerous potential advantages in the fundamental aspects of basic life support and defibrillation (BLSD). These include immediate recognition of the OHCA by the operator, eye and sound contact of the victim with the emergency system, the start of high-quality CPR, and the use of AEDs by ordinary people on site as soon as available [3].

The concept of drones, or UAVs, dates back to the First World War, where large radio-controlled planes were used as target practice for battleships [4]. These drones resembled fixed-wing aircraft and were further developed to encompass military needs for intelligence, reconnaissance, artillery, and target acquisition [4]. 

Present-day drones are versatile tools with varying take-off, landing, and payload capabilities that can be used outside the military, whether for commercial, personal, or professional use. The most commonly used commercial drones, resembling helicopters, have vertical take-off and landing (VTOL) capabilities. These typically have a shorter range, slower speed, and smaller payload capacity when compared to the horizontal take-off and landing drones, resembling fixed-wing aircraft. There is still an extensive range of capabilities within the VTOL class, such as target acquisition or infrared cameras, audio communications, payload delivery, automatic landing, and collision avoidance [5]. VTOL can hover, making them ideal for urban areas or environments where extensive movement is limited or increased maneuverability is required [6]. The possible applications of drones to medicine are extensive; however, this field is nascent, and the global use of this technology is unknown.

## Technical report

The development of our own UAV was named the PHOENIX (a direct reference to the legendary bird of Greek mythology, which when it died, after some time was reborn from its own ashes) and was designed with the aim of offering an aircraft capable of being remotely controlled for long distances, lifting up to 15 kg, having a camera with a wide-angle lens, speakers, and microphones for verbal communication with victims and people close to victims and a plastic case with first aid supplies such as an AED. These choices of materials, design, and operational functions are designed to offer a quick first response to an OHCA. This section discusses these considerations by focusing in (1) AEDs and PHOENIX and (2) the sequence of BLSD.

AEDs and PHOENIX

Mortality from SCA increases by 7%-10% with every minute delay in defibrillation [7]. Therefore, shortening the amount of time that a victim spends without defibrillation is critical to survival. Boutilier et al. (2017) examined the feasibility of creating a drone delivery network for AEDs in Toronto, Ontario, Canada. A mathematical model was applied to the 53,702 OHCAs within the eight response regions to determine the optimal placement of standing drones (bases) [8]. This study model determined that 81 bases and 100 drones were required to reduce response time by three minutes (prior to paramedic arrival) [8]. In rural settings, the response time could be reduced by as much as 10.5 minutes. When the eight regions were combined, the number of required bases reduced by 39.5% and 30% fewer drones were required to achieve similar response times, potentially reducing operational costs [8]. This confirmed that using UAVs for AEDs delivery and other time-critical medical supplies (such as epinephrine autoinjectors) would be beneficial.

Further, a simulation of drone AEDs delivery was performed in Sweden [9], with positive results. A 911 dispatcher deployed a drone, which delivered an AED to an accessible site near the scene of a cardiac arrest. The participants reported that this was a safe and feasible delivery of an AED. A potential limitation to this method was that there was a period of up to 94 seconds where the victim did not receive chest compressions due to the time required to retrieve the AED; however, in a two-person rescue scenario, there was no such interruption in compressions [9].

Finally, Cheskes et al. (2020) combined the premise of the mathematical model and simulation with a trial of delivery of AEDs by drone to two rural communities in Southern Ontario, Canada [10]. Both the drone and paramedics were dispatched at the same time, from either the same base, different bases, or with the drone in an optimized location. The time to apply an AED to a victim of cardiac arrest decreased by 1.8-8.0 minutes when a drone was utilized [10]. These three studies show a natural progression from the optimization of drone stations [8], the safety and practicality of drones for medical supply delivery at the scene [9], to the application of drones versus standard practice [10].

**Figure 1:**
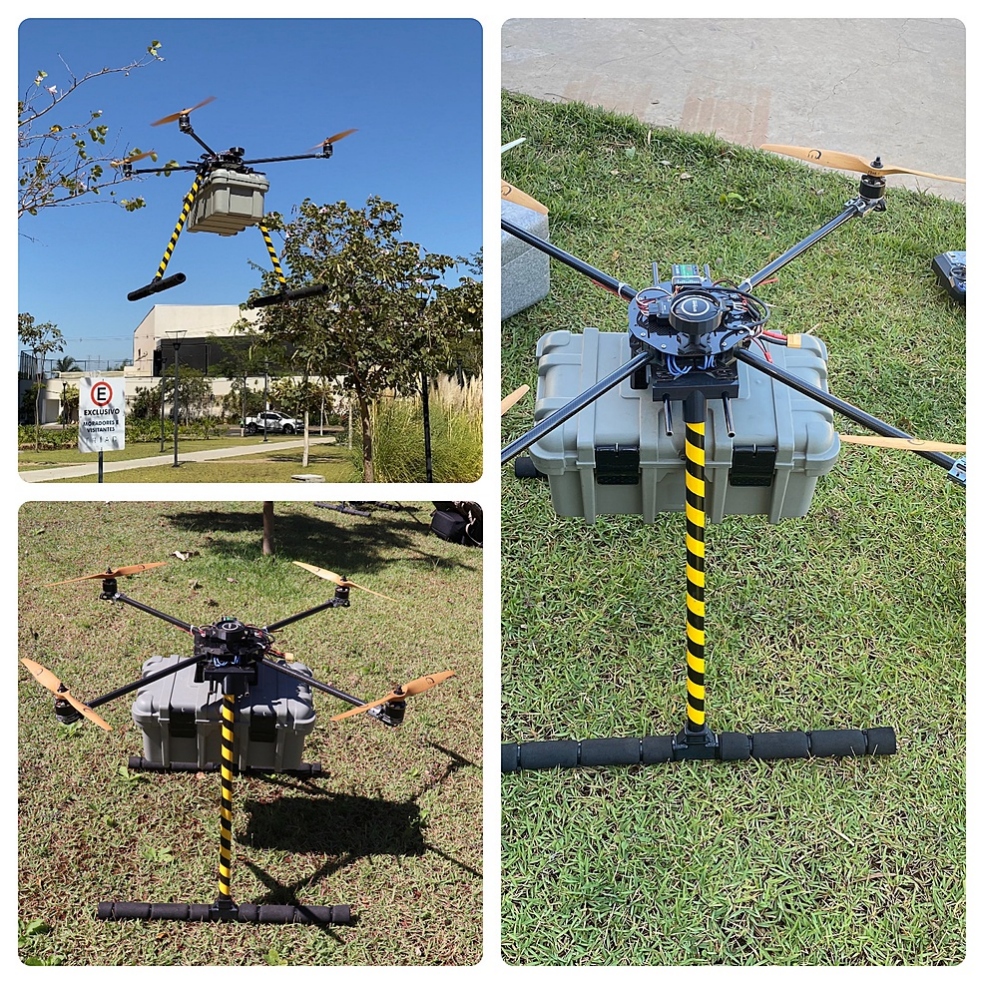
The PHOENIX Source: The authors.

Thinking about developing a scientific study and using a drone created by our team for training and simulation of care for OHCA victims, we built the PHOENIX. The construction of a fully customized drone was chosen for the production of PHOENIX due to its cost-effectiveness, ease of use, and simple assembly. Figure 1 shows our fully functional PHOENIX custom drone.

Each PHOENIX component was purchased, fabricated from commonly available commercial material, or printed using a 3D printer (see Appendix 1, Table 4).

Operational Considerations

SCA is a cardiovascular condition with high prevalence, incidence, and mortality [1]. The creation of international protocols allowed the standardization and organization of medical care around the world. Early recognition of triggering causes, effectively guiding therapeutic intervention, with an emphasis on care after the return of spontaneous circulation, brought improvements in results, contributing to a better prognosis for patients.

The PHOENIX with an AED must be used by an operator who is licensed in Canadian territory together with a healthcare professional who will guide people who have witnessed cardiac arrest victims through the 911 emergency telephone service. In addition to PHOENIX, the health professional will request the immediate dispatch of an ambulance to the location. The drone will provide medical assistance with an AED to the victim of the OHCA until help arrives by land. The successful operation of the PHOENIX, therefore, incorporates seven sets of skills [3] shown in Table 1:

**Table 1:** Skills OHCA: out-of-hospital cardiac arrest, SCA: sudden cardiac arrest, CPR: cardiopulmonary resuscitation, BLS: basic life support, AEDs: automated external defibrillators.

1. Recognition of OHCA by telephone:	The success depends on immediate and prompt recognition of an OHCA, and taking into account the new concept of SCA, such as the presence of gasping and/or the absence of a carotid pulse.
2. Implementation and training:	The training of health professionals and laypersons in the initiation of CPR is fundamental for the successful application of BLS skills.
3. Communication during OHCA:	Communication between participants during an OHCA is important for improving resuscitation maneuvers and easy recognition of errors during medical care.
4. Emphasis on CPR:	CPR should be a highlight, reinforcing the need for cardiac compressions between 100 and 120 per minute, with minimal interruptions and visualizing complete recoil of the chest until the arrival of the drone with the AEDs.
5. Creation of rapid response medical emergency team systems:	Teams formed by health professionals summoned by ambulances immediately to the site of care on suspicion of cardiac arrest.
6. Improvement in the structures of health systems:	Improvement or creation of an integrated system with the training of health professionals. In addition to the immediate availability of AEDs by drones in remote locations in concert with humans.
7. Postcardiac arrest care:	Emphasis on the neurological assessment of the patient, compliance with hemodynamic goals such as the return of spontaneous circulation, and in addition the thermal control and prevention of fever.

The fundamental aspects of BLSD in adults include immediate recognition of OHCA, contact with the emergency system, initiation of high-quality CPR, and use of the AED as soon as available [3]. Here, we will address the cardiopulmonary resuscitation performed by the layperson and health professionals, in view of the high prevalence of OHCA.

The sequence of BLSD

Chest Compressions

The main aspects to be highlighted in chest compressions are the frequency of compressions, the depth, the recoil of the chest after each compression, and minimal interruptions. For adequate tissue oxygenation, it is essential to have as few interruptions as possible during chest compressions. The chest compression fraction (CCF) is recognized as the proportion of time chest compressions performed during BLS [11]. Minimal interruptions maximize tissue perfusion and thus increase the chance of survival. For this reason, it is recommended that the pauses of chest compressions are minimized, so that the CCF is at least 60% and, ideally, 80% [12].

Breathing

Ventilations should be applied after 30 chest compressions during cardiac resuscitation, following the Compressions-Airway-Breathing (C-A-B) sequence of the Advanced Cardiovascular Life Support (ACLS) protocol. However, the priority is chest compressions and this is due to the need to generate blood flow (C) and also to avoid the practical delays inherent to attempts at adequate ventilation, mainly performed by laymen or when there is only one health professional performing assistance.

To try and establish/maintain a patent airway such as the head tilt and chin lift maneuver or the jaw angle elevation maneuver, passive ventilation occurs during chest compressions. For this reason, regardless of the technique used to apply ventilation, it is necessary to open the airway to keep it patent [13].

Ventilations through bag mask valve systems should be performed at a rate of 30 chest compressions for every two breaths, lasting only one second each. This should provide enough air to achieve complete chest elevation [14]. Hyperventilation should be avoided for these cases, as this can cause an increase in intrathoracic pressure, decreasing cardiac output and preload, and thus compromising the patient's survival [15].

Defibrillation

Early defibrillation is the treatment indicated for pulseless ventricular tachycardia (VT) and ventricular fibrillation (VF), in victims who suddenly had a cardiac collapse in an out-of-hospital setting. These are the primary cardiac dysrhythmias of an SCA in these locations [16]. Defibrillation can be performed with manual equipment by a trained physician or through the use of AEDs by both professionals and laypeople, the latter of which can be used by anyone, as quickly as possible.

**Figure 2:**
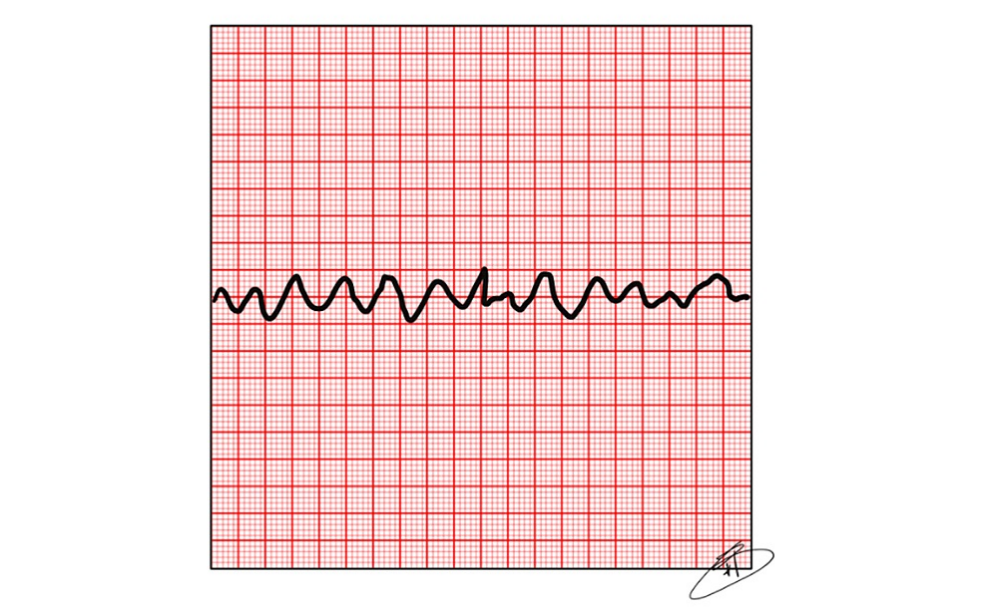
Ventricular Fibrillation (VF) Source: The authors.

**Figure 3:**
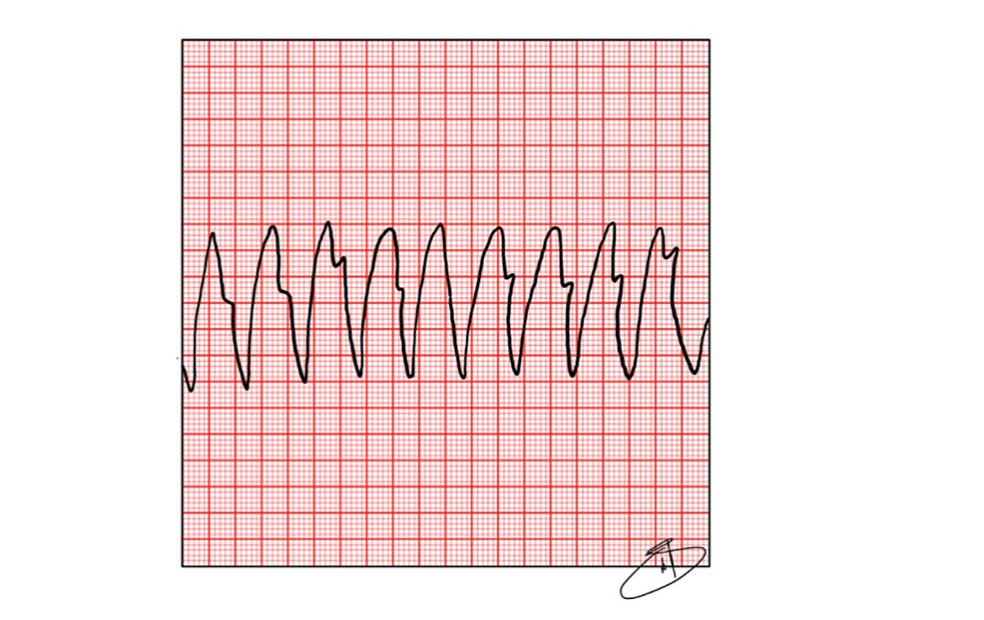
Pulseless Ventricular Tachycardia (VT) Source: The authors.

In the first five minutes of an SCA, where there are VF or VT (Figures 2, 3), the heart is more responsive to shock with a defibrillator. Beyond this time post-SCA, the amplitude of this electrocardiographic state decreases due to the depletion of the energetic substrate of the cardiac muscle. For this reason, the ideal time to deliver the “first shock” using a defibrillator, manual or AED, is in the first three to five minutes of cardiac arrest [17].

**Figure 4:**
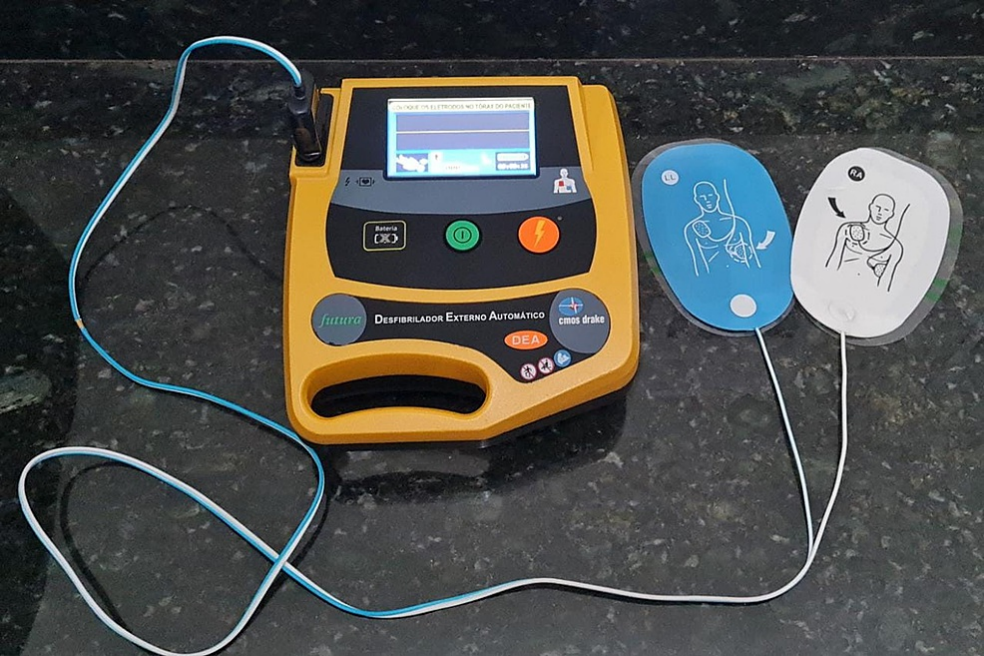
Automated External Defibrillator (AED) Source: The authors.

The AED is a small, compact, and portable device, capable of interpreting the electrocardiographic wave for the treatment with “electric shock”. By pushing a single button, the AED automatically selects the energy level and charges the electric charge in Joules automatically, leaving the operator only to press the “shock button” when indicated by means of a voice command or by light prompts (Figure 4).

The AED should be used as early as possible after cardiac arrest begins. It is recommended that chest compressions in CPR be provided, while the pads are being applied and until the AED is ready to analyze the rhythm [17].

As soon as the AED is delivered to the operator or rescuer, if he or she is alone, he or she must stop chest compression to connect the device to the patient. However, if there is more than one rescuer, the second rescuer handles the defibrillator, and, in this case, the compressions will only be interrupted when the device emits an audible alert such as “analyzing the heart rate”, “do not touch the patient!” and/or “Recommended shock, carrying, move away from the patient!”.

In an SCA situation, a mnemonic can be used to describe the simplified service steps: the “C-A-B-D” where "C" corresponds to Compressions (30 compressions), "A" is the opening of the Airways, "B" refers to Breathing (two ventilations), and "D" refers to Defibrillation with the AEDs [18].

## Discussion

Simulation scenario

Prescenario

The following is presented to the participants as a stem of the simulation case: “You are part of a rural community with a cottage hospital located in Newfoundland and Labrador, Canada, where there are a physician who answers the phone, a licensed drone pilot, a PHOENIX drone capable of providing an AEDs, and a team of paramedics with an ambulance. The sky is clear and without strong winds, but the night before it snowed a lot. At 12:10, the phone rings and a 25-year-old woman is crying while asking for help. She is camping with her 55-year-old father in a park 8 km from the city, and she reports that he is on the ground and not breathing.”

His daughter reports that approximately three minutes ago, the patient started experiencing severe chest pain and fell to the ground. He does not respond to any painful or verbal stimuli.

This simulation scenario is based on presumed OHCA, and the entire simulation will be based on the necessary care pertinent to this information. The main areas of expertise are guidance about safety; guide assess the victim's responsiveness; send help (drone and ambulance); guide to check breath and pulse; and guide start cycles of 30 compressions and two ventilations. These learning objectives are shown in Table 2.

**Table 2:** Learning Objectives AED: automated external defibrillator.

Learning objectives
Guidance about safety: The participants must ensure that the site is safe for the rescuer and the victim so that he or she does not become the next victim. If the location is safe, then care of the patient can safely continue.
Guide assessing the victim's responsiveness: The participants should guide how the victim's responsiveness should be assessed by calling out and shaking their shoulders. If the victim responds, participants should guide the rescuer to talk to the victim and ask if he or she needs help. If the victim does not respond, immediately send the drone and ambulance.
Send help (drone and ambulance): In an extra-hospital environment, participants must immediately send the PHOENIX with an AED and an ambulance with a team of paramedics. At the same time, participants must advise on resuscitation maneuvers. It is important to designate people to be responsible for performing these functions.
Guide to check breathing and pulse: If the victim does not breathe or gasps and the pulse is absent, participants should promptly initiate cardiopulmonary resuscitation.
Guide start cycles of 30 compressions and two ventilations: Participants should guide the start cycle of 30 compressions and two ventilations, considering that there is a barrier device (for example, a pocket mask to apply the ventilation).

Inputs

Equipment

This simulation can be performed in a controlled environment using a mannequin or an actor. A camera on a tripod, speakers, and microphones can play the role of a drone at the service point in addition to an AED.

This simulation is intended for health professionals who operate within an urgent or emergency network such as 911 where there is a trained healthcare professional who can assist and guide patients and victims by telephone. This simulation will be performed in both urban and rural or remote places where a drone reaches the victim first but a road ambulance is delayed. Recommended equipment is listed in Appendix 2, Table 5.

Information

Ideally, the simulation is aimed at healthcare professionals who work in emergency care such as 911 in urban or rural areas or who seek additional training in the treatment of patients with cardiac arrest. As the simulation takes place in a prehospital environment, there is no access to imaging or laboratory investigations. 

Facilitators

Two health professionals, both comfortable in providing care for patients with SCA, should act as facilitators. These professionals must have experience with the use of AEDs. One should be designated as the primary facilitator, guiding participants and helping with the overall organization and execution of the case, while the second facilitator will be present to assess individual performance. Facilitators are responsible for providing participants with the appropriate information as requested. Facilitators should examine the scenario in advance to identify possible limitations or technical problems related to the proper functioning of the camera, speakers, microphone, and AEDs.

Context

Participants initiate the case in a 911 urgent and emergency care service, which contains a physician and a licensed drone pilot (the latter can be interpreted by the facilitator). The phone rings and a 25-year-old woman is desperate and crying a lot on the phone. She is camping with her 55-year-old father and reports that three minutes ago he screamed with a pain in his chest and simply fell to the ground. She believes that he is not breathing.

Prebriefing

Before starting the simulation, participants must be submitted to the fiction contract, which recognizes that everything taking place during the simulation must be treated as if they were "real" so that the objectives of the simulation can be achieved. During this period, the facilitators present the simulation scenario and all the necessary precautions, before presenting the AED, the simulation scenario with the camera, speakers, and microphones, and describe the function of each piece of equipment. If there is a limited supply of participants, the paramedic’s team can only be informed by the facilitators, as the main objective of this simulation is the fundamental aspects of BLSD in adults, which include immediate recognition of SCA, contact with the emergency system, initiation of high-quality cardiopulmonary resuscitation, and use of AED as soon as possible. 

Table 3 shows the objectives checklist of the simulation in detail. The scenario begins with the patient on the ground, with the rescuer beside the victim and using the smartphone to call for help.

**Table 3:** Objectives Checklist Y: yes, N: no; VF: ventricular fibrillation, VT: ventricular tachycardia, AED: automated external defibrillator.

Expected action	Findings/Justification	Completed (Y/N)
1. Take the phone call and identify yourself as a doctor.	A woman is crying and asking for help: The participant must collect information from the woman on the phone about the victim and the scene.	
2. Guidance about safety.	If there are risks to the lay rescuer by scene or unsafe environments: If possible and without offering greater risks to the lay rescuer, remove the patient to a safer place as soon as possible.	
3. Guidance on assessing the victim's responsiveness and breathing.	The victim does not respond: The victim does not respond and help should be sent immediately to the scene.	
4. Sending help!	The participant requests that the drone and ambulance be sent: The participant quickly identified a cardiac arrest and immediately sent help.	
5. Guide to check breathing and pulse: Guidance on checking the pulse and starting cardiopulmonary resuscitation if the pulse is absent or if he or she is in doubt.	The lay rescuer does not know if there is a central pulse: The victim still does not respond to any stimulus.	
6. Guide to start cycles of 30 compressions and two ventilations: Guide the completion of three cycles of continuous compressions (200) with passive oxygen ventilation, in cases of cardiac arrest witnessed with VF/VT rhythm. I: Guide about the positioning of the hand: Rock the heel of the hand off the chest, keeping fingertips on the chest wall to maintain hand position. II: Chest compressions must have a minimum depth of 5 cm, without exceeding 6 cm. III: The delay of compressions between shock delivery must be as short as possible. IV: Resume chest compressions immediately after defibrillation for adults in cardiac arrest.	Wait for AED guidance: 1) “analyzing the heart rate”; or 2) “do not touch the patient”; and/or 3) “recommended shock, charging, move away from the patient”.	
7. Performing 30 compressions and two ventilations, for adults in cardiac arrest. I: It is advisable to perform compressions at a frequency of 100 to 120 compressions/minute. II: Chest compressions must have a minimum depth of 5 cm, without exceeding 6 cm. III: The delay of compressions between shock delivery must be as short as possible. IV: Resume chest compressions immediately after defibrillation for adults in cardiorespiratory arrest.	Wait for AED guidance: 1) “analyzing the heart rate”; or 2) “do not touch the patient”; and/or 3) “recommended shock, charging, move away from the patient”.	
8. Guide to maintain the cardiopulmonary resuscitation maneuvers and the AEDs analysis until the paramedic team arrives at the scene and takes over or until the victim shows signs of life.	The paramedic team has arrived or not at the scene and will take over the case: The participant must instruct the lay rescuer to let the paramedic team take over the scenario.	
9. Guide to maintain the cardiopulmonary resuscitation maneuvers and the AEDs analysis until the paramedic team arrives at the scene and takes over or until the victim shows signs of life.	The victim woke up and started to move. The participant must instruct the layperson to stop the cardiac compressions, disconnect from AED machine, and wait for the paramedic's team to arrive at the location.	
10. The participant must now instruct the woman that her father will be taken to a hospital by the paramedic team.	The paramedic team reports that the 55-year-old man returned with a central pulse and is demonstrating spontaneous breathing.	

Debriefing 

After completing the simulation scenario, instructors will guide a debriefing session allowing reflection of key points of the simulation scenario. All learners will now have the opportunity to take a moment to highlight whether their experience was positive or negative. This time will be dedicated to a brief reflection of the case, and the learners will have the opportunity to ask questions about the simulation itself, focusing on the objectives established in the simulation scenario. Learners should also be encouraged by instructors to provide feedback on using drones for the medical field and running this simulation. The debriefing should be facilitated by the two trainers who should provide constructive feedback to each participant and should follow an advocacy-inquiry as an appropriate model for the debrief [19].

During the debrief, instructors must also set aside a specific time of didactic instruction to provide students with additional information related to learning objectives in clinical cases involving SCA. The inclusion of a didactic session should last approximately twice as long as the simulation session. This should be done immediately after training so that learners can consolidate key clinical information and highlight knowledge gaps for future learning [20]. The main teaching points to be covered in the didactic session are included in Table 1.

Discussion

The purpose of this technical report was two-fold. First, we describe the potential benefits of drones for application in the medical field as an out-of-hospital cardiac arrest training device. Second, we describe a simulation scenario focusing on the key role of practice and assessment of communication skills that can be used to train healthcare professionals in how, through contact with laypersons using a camera and microphone connected to a drone, to immediately identify an SCA victim and then quickly arrange care for that victim by sending a drone with an AED and a team of paramedics by land.

Our scenario helps healthcare professionals become familiar with the use of AEDs in terms of safety, operations, and instruction given to laypeople. Although the scenario is based on out-of-hospital cardiac arrest care, it can be adapted to in-hospital settings with the use of manual defibrillators.

## Conclusions

Simulation-based training, as proposed in this article, can allow health professionals to improve their knowledge and techniques on communication and instruction to laypersons in the proper use of AEDs. The guidance of the emergency system attendant can increase the layperson's performance in the out-of-hospital cardiac arrest care, guiding him/her in identifying the victim's breathing, using a defibrillator, and how to perform chest compressions.

When defibrillation is performed in less than five minutes of SCA, the survival potentially can approach 50% to 70%. We present the use of a drone that carries an AED with visual and verbal communication systems and, in addition, is able to reach the location where the victim can be quickly offered early defibrillation. This has the potential to contribute to the reduction of mortality rates for victims of out-of-hospital cardiac arrest.

## Appendices

Appendix 1

**Table 4:** PHOENIX Components ESC: electronic speed controller, APM: Ardupilot Mega, GPS: Global Positioning System, ABS: Acrylonitrile-Butadiene-Styrene.

Item	Brand	Amount
Engine 710 Kv	T-Engine	4
ESC 30A		4
Propeller 13 x 6.5	Xoar	4
Locking nut 6 mm		4
Flat washer 6 mm		4
Aluminum engine base		4
Base screw 3 mm		8
Motor screw 3 mm		16
Carbon tube 14 mm x 12 mm x 32 mm		4
Aluminum tube base		4
Allen screw base 3 mm		16
Round carbon base 15 mm x 2 mm		2
Flight controller APM 2.8	Ardupilot	1
GPS M8N		1
Base 3D ABS p GPS		1
Receiver 10Ch	Flysky	1
Cable jumper 3 ways		4
Power modulo	3Dr	1
Carbon base for coupling		2
Carbon tube 8 mm x 6 mm x 33 mm		2
Nylon clamp		20
Insulating tape		1
Aluminum tube 20 mm x 19 mm x 50 mm		4
Base 3D ABS p Tubos 20 mm		2
Ethyl, vinyl, and acetate (EVA) for support		4
3D ABS T connector for support		2
Radio 10ch	TurniGy	1
Electric battery 18650		2
Case for electric battery 3D ABS		1
Antenna 5Dbi		2
Step down		1
Plastic case		1
GoPro camera		1
Speakers		1
Microphone		1

Appendix 2 

**Table 5:** Recommended Equipment AED: automated external defibrillator.

Resource	Description
One GoPro camera	This equipment will be used to simulate the drone's camera for real-time visual monitoring of the victim's situation.
One speaker	This equipment will be used to simulate drone’s speakers so that the rescuer can hear the regulator’s instructions.
One microphone	This equipment will be used to simulate the microphones of the drone so that the medical regulator can listen to the rescuer in real time.
A mannequin	This simulator will be used to simulate a cardiac arrest victim.
A computer	This equipment will be used to simulate the drone controller in real time.
One AED	This equipment will be used to simulate cardiac arrest treatment in which the patient would be presenting ventricular tachycardia or ventricular fibrillation.
